# Socioeconomic disparities in orthodontic treatment outcomes and expenditure on orthodontics in England’s state-funded National Health Service: a retrospective observational study

**DOI:** 10.1186/s12903-017-0414-1

**Published:** 2017-09-19

**Authors:** Juliet Price, William Whittaker, Stephen Birch, Paul Brocklehurst, Martin Tickle

**Affiliations:** 1 Division of Dentistry, School of Medical Sciences, University of Manchester, Manchester, UK; 2 Manchester Centre for Health Economics, School of Health Sciences, University of Manchester, Manchester, UK; 3 Department of Clinical Epidemiology and Biostatistics, McMaster University, Hamilton, Canada; 4 Institute of Medical and Social Care Research, Bangor University, Bangor, UK; 5 Division of Dentistry, School of Medical Sciences, University of Manchester, Manchester, UK

**Keywords:** Orthodontics, Malocclusion, Treatment outcomes, Socioeconomic status

## Abstract

**Background:**

This study aimed to assess whether there are potential areas for efficiency improvements in the National Health Service (NHS) orthodontic service in North West England and to assess the socioeconomic status (SES)-related equity of the outcomes achieved by the NHS.

**Methods:**

The study involved a retrospective analysis of 2008–2012 administrative data, and the study population comprised patients aged ≥10 who started NHS primary care orthodontic treatment in North West England in 2008. The proportions of treatments that were discontinued early and ended with residual need (based on post-treatment Index of Orthodontic Treatment Need [IOTN] scores that met or exceeded the NHS eligibility threshold of 3.6) and the associated NHS expenditure were calculated. In addition, the associations with SES were investigated using linear probability models.

**Results:**

We found that 7.6% of treatments resulted in discontinuation (which was associated with an NHS annual expenditure of £2.3 m), and a further 19.4% (£5.9 m) had a missing outcome record. Furthermore, 5.2% of treatments resulted in residual need (£1.6 m), and a further 38.3% (£11.6 m) had missing IOTN data (due to either a missing outcome record or an incomplete IOTN outcome field in the record), which led to an annual NHS expenditure of £13.2 m (44% of the total expenditure) on treatments that are a potential source of inefficiency. Compared to the patients in the highest SES group, those in the lower SES groups were more likely both to discontinue treatment and to have residual need on treatment completion.

**Conclusions:**

Substantial inefficiencies were evident in the NHS orthodontic service, with 7.6% of treatments ending in discontinuation (£2.3 m) and 5.2% ending with residual need (£1.6 m). Over a third of cases had unreported IOTN outcome scores, which highlights the need to improve the outcome monitoring systems. In addition, the SES gradients indicate inequity in the orthodontic outcomes, with children from disadvantaged communities having poorer outcomes compared to their more affluent peers.

**Electronic supplementary material:**

The online version of this article (10.1186/s12903-017-0414-1) contains supplementary material, which is available to authorized users.

## Background

In England, NHS expenditure on the primary care orthodontic service amounts to approximately £250 m per year [1, 2], which makes ensuring maximum value for money in the NHS orthodontic service critical. Moreover, the expenditure may continue to increase as a result of increases in social acceptance of fixed orthodontic appliances and expectations regarding dental appearance [3, 4]. However, the NHS is currently under a great deal of pressure to reduce expenditure because NHS commissioners and providers ended 2015/2016 with an aggregate deficit of £1.85 billion (a threefold increase on the previous year) [5].

A number of studies, which were largely hospital-based studies, have highlighted the potential for suboptimal outcomes in the NHS orthodontic service in England and Wales. These studies have focused on both discontinuation (i.e., failure to complete a course of active treatment) [6, 7] and suboptimal outcome scores based on occlusal indices such as the Index of Orthodontic Treatment Need (IOTN) [8], the Index of Complexity, Outcome, and Need (ICON) [8, 9], and the Peer Assessment Rating (PAR) index [8, 10] (the PAR index was developed as a simple tool for assessing the orthodontic outcomes of groups of patients, rather than individual patients and, as part of the NHS orthodontic outcome monitoring system, providers are required to report PAR scores for 20 patients plus 10% of the additional patients [11]).

In addition, several studies have explored the associations between socioeconomic status (SES) and suboptimal orthodontic outcomes in the NHS. For example, a hospital-based study in England of 144 orthodontic patients aged between 9 and 19 reported that SES was not associated with discontinuation [6]. In contrast, a study based on 1990–1991 Dental Practice Board data from England and Wales reported a positive association between deprivation and discontinuation [7]. Moreover, a hospital-based study in England found a positive association between deprivation and ‘low or no improvement in occlusion’ (defined as a score lower than the sample median ICON improvement score of a modified version of the ICON), based on an analysis of 135 12- to 16-year-olds treated for 1 year with fixed orthodontic appliances [9].

In this study, we aimed to identify the scale of potential efficiency improvements in the NHS primary care orthodontic service. To do this, we quantified the proportion of, and NHS expenditure on, treatments that were discontinued early and those that ended with residual post-treatment need. Secondly, we aimed to analyse whether the proportions of treatments ending in discontinuation and residual need differed by SES in order to assess the equity of the outcomes achieved by the NHS and whether initiatives to improve the efficiency of the service should be targeted at specific populations.

## Methods

The study involved a retrospective analysis of data from 1 January 2008 to 31 December 2012 from all orthodontic activity records (i.e., FP17O records) submitted by primary care dentists who were working under state-funded NHS contracts in North West England [12]. The pseudo-anonymised data set was provided by the National Health Service Business Services Authority (NHSBSA).

The study population comprised patients ≥10 years old who started orthodontic treatment in 2008. The analysis was restricted to patients aged ≥10 because those aged ≤9 receive only interceptive treatment (which amounted to 1.6% of the treatments in the data set), while those aged ≥10 receive full courses of treatment. The NHS provides the vast majority of orthodontic treatments for children in the UK, with only 2.4% (95% CI: 0.7–4.0%) of children having private treatment by the age of 15 [13]. As the data set used in this study represented the entire population of patients aged ≥10 who received orthodontic treatment under state-funded NHS contracts in North West England, inferential statistics were unnecessary.

The data set included clinician-reported information on whether each treatment was completed or discontinued, along with IOTN outcome scores that were used to calculate residual need (these are assessed at the final appointment before treatment completion or discontinuation).

Discontinuation was defined as failure to complete a course of active treatment before the end of the treatment plan (as recorded by clinicians in the FP17O records). Discontinuation can be initiated by patients (who can request to stop treatment early or fail to return for treatment; once it has been established that a patient does not wish to return, the dentist is required to submit an outcome record) or by dentists (e.g., when a patient fails to comply with oral hygiene advice, repeatedly misses appointments, or breaks their orthodontic appliance) [12].

Residual need was measured based on whether each patient’s IOTN score at the end of active treatment met or exceeded the NHS IOTN eligibility threshold of 3.6 (i.e., a Dental Health Component [DHC] score of 3 with an Aesthetic Component [AC] score of 6) [14]. Discontinuation and residual need are not mutually exclusive in that, for example, a patient can discontinue treatment (e.g., because the patient’s parents can no longer take their child to orthodontic appointments) and yet have no residual need by the time they discontinue (despite not completing the full planned course of treatment).

As self-assessment of treatment outcomes can be subject to bias, providers are encouraged to utilise the services of an independent third party calibrated in the use of the IOTN and the PAR index [15]. In addition, the NHSBSA carries out independent monitoring of five patients of 450 orthodontic clinicians per year, which involves requesting full patient records, including photographs, radiographs, and pre- and post-treatment models of the patients’ occlusions [16]. While this monitoring process often involves flagging up issues for discussion with the orthodontic clinicians (which are then resolved without further scrutiny), it very rarely leads to further investigation being conducted [16].

Firstly, we calculated the proportions of patients who a) discontinued treatment, b) completed treatment, and c) had a missing outcome record (i.e., no record submitted within the study period indicating that treatment had been completed or discontinued). The analysis was restricted to treatments that started in 2008, treatment usually takes 18–24 months to complete [17], and the data set provided outcomes for the subsequent 4 years, so missing outcome records were considered to be reflective of reporting issues.

Secondly, we calculated the proportions of patients with residual need, no residual need, and incomplete IOTN outcome fields among a) patients who discontinued treatment and b) those who completed treatment. Thirdly, we calculated the overall proportions of patients who had residual need, no residual need, incomplete IOTN outcome fields, and missing outcome records.

NHS expenditure was determined by calculating the numbers of Units of Orthodontic Activity (UOAs) carried out in 2008 by the NHS orthodontic providers. Under NHS payment arrangements, the quantity of services that an orthodontic provider is contracted to deliver is expressed on the basis of an annual target number of UOAs (as agreed by the NHS and the provider), and the annual payments are issued as monthly instalments, 1 month in arrears [1]. Courses of treatment (including an initial assessment) for patients aged 10–17 attract 21 UOAs and those for patients aged >17 attract 23 UOAs; one UOA (the value of which differs between different contracts) has a mean value of approximately £59 [1].

To assess whether the proportions of treatments ending in discontinuation and residual need differed by SES, ordinary least squares (OLS) linear regressions were used (whilst not used to generate statistical tests, this approach provided details on the proportions of children with the treatment outcomes and enabled our analyses to be adjusted for potential confounding). The data set included area-level SES data in the form of Index of Multiple Deprivation (IMD) scores [18] that had been matched by the NHSBSA to the patients’ postcodes at the level of Lower-layer Super Output Areas (LSOAs). Cut-points for IMD quintiles (with respect to the population of England) were used to calculate each patient’s IMD quintile. The first SES analysis assessed whether discontinuation differed by SES, and the second and third analyses assessed whether residual need differed by SES for patients who discontinued and completed treatment, respectively.

Initially, unadjusted SES analyses were carried out (i.e., only the SES dummy variables were included in the models). Subsequently, the analyses adjusted for potential confounding (to separate the effect of confounders from the effect of SES) by adding the following potential confounders to the models: IOTN AC score at the start of treatment (1–10; which was used to reflect the complexity of treatment), gender, and age. This helped to ensure that any SES-related differences would not reflect, for example, differences in treatment complexity or late uptake of treatment.

In each model, the SES coefficients represent the mean percentage-point difference (compared to being in the reference category, which was the least deprived group) in the proportion of children with the treatment outcome variable (discontinuation or residual need) when all other variables besides SES (i.e., the potential confounders) are held constant.

To assess potential biases to the SES analyses due to incomplete IOTN outcome fields and missing outcome records, we explored whether the number of incomplete IOTN outcome fields differed by SES (for patients who discontinued and completed treatment) and whether the number of missing outcome records differed by SES.

The statistical analyses were conducted using Stata version 13 [19].

## Results

There were 24,501 treatment starts in 2008. Of these, 4746 (19.4%) were missing an outcome record, while the remaining 19,755 (80.6%) had an associated outcome record. Of the 19,755 outcome records, 4636 (23.5%) had incomplete IOTN outcome fields (3456 for patients who completed treatment and 1180 for those who discontinued treatment).

As shown in Table 1, 7.6% of treatments were discontinued and 73.1% were completed, while the remaining 19.4% had missing outcome records. Among the treatments associated with outcome records (*n* = 19,755), 9.4% were discontinued and 90.6% were completed. The proportion of patients with residual need among those who discontinued and completed treatment was 16.3% and 5.4%, respectively.

**Table 1:** Treatment outcomes associated with discontinuation

Outcome^a^	Percentage (relative frequency)			NHS expenditure (GBP)
	All *n* = 24,501	Males *n* = 10,571	Females *n* = 13,930	
Discontinuation	7.6 (1856/24501)	9.0 (951/10571)	6.5 (905/13930)	2,300,764
Residual need	16.3 (303/1856)	14.4 (137/951)	18.3 (166/905)	
No residual need	20.1 (373/1856)	20.6 (196/951)	19.6 (177/905)	
Incomplete IOTN	63.6 (1180/1856)	65.0 (618/951)	62.1 (562/905)	
Completion	73.1 (17,899/24501)	71.6 (7574/10571)	74.1 (10,325/13930)	22,186,773
Residual need	5.4 (961/17899)	5.7 (431/7574)	5.1 (530/10325)	
No residual need	75.3 (13,482/17899)	74.8 (5662/7574)	75.7 (7820/10325)	
Incomplete IOTN	19.3 (3456/17899)	19.6 (1481/7574)	19.1 (1975/10325)	
Missing outcome record	19.4 (4746/24501)	19.4 (2046/10571)	19.4 (2700/13930)	5,890,206

*GBP* Great Britain Pound, *IOTN* Index of Orthodontic Treatment Need, *NHS* National Health Service

^a^An outcome could be specified as either treatment discontinuation or treatment completion

As shown in Table 2, overall, 5.2% of patients had residual need and 56.5% had no residual need, while the remaining 38.3% had either incomplete IOTN outcome fields (18.9%) or missing outcome records (19.4%). Among the treatments with IOTN outcome scores (*n* = 15,119), 8.4% had residual need and 91.6% had no residual need. Compared to female patients, male patients were more likely, overall, to discontinue treatment and to have residual need.

**Table 2:** Treatment outcomes associated with residual need

Outcome	Percentage (relative frequency)			NHS expenditure (GBP)
	All *n* = 24,501	Males *n* = 10,571	Females *n* = 13,930	
Residual need	5.2 (1264/24501)	5.4 (568/10571)	5.0 (696/13930)	1,567,866
No residual need	56.5 (13,855/24501)	55.4 (5858/10571)	57.4 (7997/13930)	17,172,127
Incomplete IOTN outcome field	18.9 (4636/24501)	19.9 (2099/10571)	18.2 (2537/13930)	5,747,544
Missing outcome record	19.4 (4746/24501)	19.4 (2046/10571)	19.4 (2700/13930)	5,890,206

*GBP* Great Britain Pound, *IOTN* Index of Orthodontic Treatment Need, *NHS* National Health Service

In 2008, £2.3 m was expended by the NHS on treatments in North West England for patients aged ≥10 that ended in discontinuations (Table 1). The corresponding value for treatments that were discontinued or completed with residual need was £1.6 m (Table 2). A further £11.6 m was expended on treatments that had missing IOTN outcome data (Table 2). Thus, residual need and missing IOTN outcome data was associated with an expenditure of £13.2 m (i.e., 44% of the total NHS expenditure).

Table 3 shows the treatment outcomes by SES. Discontinuation was more likely for those in the lower SES groups compared to those in the highest SES group, and this association persisted after adjusting for potential confounders (Model 1); the distributions of the potential confounders by IMD quintile are provided in Additional file 1. Furthermore, among the patients who completed treatment, those in the lower SES groups were also more likely to have residual need than those in the highest SES group, and this association persisted in the adjusted analysis (Model 2). However, among the patients who discontinued treatment, there was no association between SES and residual need (Model 3).

**Table 3:** Treatment outcomes by socioeconomic status (SES)

IMD quintile (reference category: 5 [least deprived])	Mean percentage change					
	Model 1: Discontinuation vs. completion *n* = 19755^a^		Models 2 and 3: residual need vs. no residual need			
			Among patients who completed *n* = 14443^b^		Among patients who discontinued *n* = 676^b^	
	Unadjusted	Adjusted for IOTN AC, gender, age	Unadjusted	Adjusted for IOTN AC, gender, age	Unadjusted	Adjusted for IOTN AC, gender, age
1	6.4	6.1	2.8	2.6	3.5	0.6
2	5.3	5.1	3.3	3.2	−1.6	−1.0
3	3.1	3.1	1.8	1.8	0.3	−0.5
4	1.2	1.1	1.0	1.0	−0.3	−1.2
Missing	3.0	3.0	0.0	−0.3	−10.9	−12.5

*AC* Aesthetic Component, *IMD* Index of Multiple Deprivation, *IOTN* Index of Orthodontic Treatment Need

^a^Only patients with outcome records could be included (4746 had missing outcome records)

^b^Only patients with completed IOTN outcome fields could be included (out of those who completed and discontinued, 3456 and 1180 had incomplete IOTN outcome fields, respectively). The same sample sizes were used for the unadjusted and adjusted analyses (as there were no missing data on gender and age, and a dummy variable was used for the missing IOTN AC data)

As shown in Table 4, higher SES was associated with incomplete IOTN outcome fields among patients who completed treatment (Model 4), but there was no association with SES among those who discontinued treatment (Model 5). In contrast, lower SES was associated with missing outcome records (Model 6). The SES-related associations persisted in the adjusted analyses.

**Table 4:** Missing outcome data by SES

IMD quintile (reference category: 5 [least deprived])	Mean percentage change					
	Models 4 and 5: Incomplete IOTN outcome field				Model 6: Missing outcome record *n* = 24,501	
	Among patients who completed *n* = 17899^a^		Among patients who discontinued *n* = 1856^a^			
	Unadjusted	Adjusted for IOTN AC, gender, age	Unadjusted	Adjusted for IOTN AC, gender, age	Unadjusted	Adjusted for IOTN AC, gender, age
1	−6.5	−6.9	0.8	0.7	3.0	3.0
2	−4.0	−4.2	−1.7	−2.3	2.4	2.3
3	−2.2	−2.4	−3.3	−3.3	1.9	1.8
4	−0.8	−0.9	−0.9	−0.5	1.9	1.9
Missing	−6.3	−6.5	2.6	1.6	0.7	1.1

*AC* Aesthetic Component, *IMD* Index of Multiple Deprivation, *IOTN* Index of Orthodontic Treatment Need

^a^Only patients with outcome records could be included (4746 had missing outcome records)

## Discussion

In North West England, 7.6% of orthodontic treatments that were started in 2008 for patients aged ≥10 resulted in discontinuation and 5.2% resulted in residual need. The proportion of patients with residual need among those who discontinued and completed treatment was 16.3% and 5.4%, respectively. It is likely that early discontinuation has a much larger effect on residual need than discontinuation near the end of a course of treatment. However, our results show that residual need is evident for some patients even when a course of treatment is completed.

NHS expenditure on treatments that resulted in discontinuation amounted to £2.3 m, and £1.6 m was expended on treatments that ended with residual need (for treatments that were either discontinued or completed). These figures highlight the need to increase the cost-effectiveness of NHS orthodontic care.

Moreover, previous studies of NHS orthodontic treatment outcomes [6–10] have tended to report higher rates of poor treatment outcomes compared to those identified in our study. For example, a study in England of 144 patients who were treated at several hospitals and a primary care practice found that 43% failed to complete their treatment (the most common reasons being poor oral hygiene, multiple missed appointments, and orthodontic appliance breakages) [6]. However, most of the patients were hospital patients, and treatment provision may also have been affected by the three orthodontic clinicians involved being aware that the discontinuation rates would be published. Another study compared the differences in residual need rates (as measured using the IOTN, PAR index, and ICON) for 130 patients who were treated in hospitals in the North of England [8]. The study found that different occlusal indices indicated differing levels of residual need, for example, 20.1% of patients had residual need according to their IOTN DHC scores and 17.2% according to their ICON scores [8]. In contrast to our study, the study involved hospital patients (as in the abovementioned study), so the sample may represent a more complex case mix than those treated in primary care practices, and the skills and training of the clinicians involved would differ from those of primary care orthodontic clinicians.

We also found that lower SES was associated with discontinuation and residual need after completing treatment, indicating SES-related inequality in outcomes. However, there was no association between SES and residual need among the patients who discontinued treatment, which suggests that ceasing treatment early does not contribute to SES-related inequality in residual need.

Previous UK studies have also indicated that there are associations between low SES and poor treatment outcomes. First, a study in England and Wales of 1431 patients (based on a 1990–1991 Dental Practice Board data set that covered all patients who discontinued treatment and 2% of those who had completed treatment) found a larger percentage of those from the more deprived groups (using multiple area-level SES measures) discontinued treatment [7]. A study in England of 135 12- to 16-year-olds treated for one year with fixed orthodontic appliances in the hospital dental service found that deprivation (based on characteristics of parental employment) was associated with high improvement in occlusion (defined as a score equal to or higher than the sample median ICON improvement score of a modified version of the ICON) [9]. However, other elements of SES, namely parental education and employment status, were not associated with treatment outcomes [9]. Moreover, a study in North West England of 144 9- to 19-year-olds reported that SES (measured using Townsend scores) was not associated with discontinuation [6]. However, this study largely involved patients treated in hospitals, who (like the patients in the abovementioned study) may have had a different treatment experience compared to those treated in primary care.

SES may be linked with discontinuation because patients in the more deprived groups have been reported to be more likely to miss orthodontic appointments [20] and discontinuation can be increased by orthodontic practices having a strict policy on discontinuing treatment for patients who miss appointments [21]. It is likely that the more deprived groups are more affected by prohibitive transport costs and the potential impact of lost pay for the accompanying parents [22]. In addition, the SES associations with treatment outcomes may be due to the effect of SES on the patient’s development of self-efficacy [23] (i.e., the strength of one’s belief in one’s ability to complete tasks and reach goals [24]). Self-efficacy influences health behaviours [25], and health behaviours (e.g., patient compliance with treatment instructions such as to regularly replace intraoral elastics) are associated with orthodontic outcomes [26–28]. Studies have shown that low SES is associated with some of the elements of low patient compliance, such as poor oral health practices [29–31].

The data set provided comprehensive individual-level data on all NHS primary care orthodontic treatments provided under NHS contracts in North West England, and the IMD score for each patient. While the results reflect orthodontic outcomes in North West England, they may not be generalizable to other populations if there are regional variations in practitioner processes and patient preferences. However, practitioner processes should largely be uniform across England given that the same standards and procedures exist across NHS England. Also, patient preferences are likely to be generalizable, and approximately 13% of individuals aged ≥10 in England resided in North West England in 2011 [32], so the analyses were conducted on a significant percentage of the population of England. Additionally, North West England has a diversity of individuals from different SES backgrounds, with all five IMD quintiles being represented (though the lower SES groups are overrepresented, with approximately a third of North West LSOAs being in the most deprived quintile in England [33]).

We used the IMD (which is an area-level measure that takes into income, employment, health, education, crime, housing and services, and living environment) because there were no individual- or household-level data on income, occupation, or other indicators of SES. The IMD is the most commonly used measure of SES in the UK; however, one limitation of the use of the IMD is that not everyone living in a deprived area is deprived and not all deprived people live in deprived areas. This implies that there can be misclassification error, which could bias the SES-related associations. IMD scores are typically reported at the LSOA level, which represents the smallest area for reporting UK census data, with population sizes of 1000–3000 individuals. As the population size of small-area deprivation measures decreases, the risk of misclassification error decreases. Although individual- or household-level SES indicators would help to avoid misclassification error, collecting individual-level data on, for example, self-reported income, increases the likelihood of non-response. In addition, despite the risk of misclassification error, small-area SES measures can help to identify areas with higher proportions of deprived households, so they are useful for planning and targeting healthcare services [34].

Another major limitation of the study is the missing outcome data, i.e., 18.9% of treatments (associated with an NHS expenditure of £5.9 m) ended without an outcome record being submitted and 19.4% were associated with incomplete IOTN outcome fields (£5.7 m). This inevitably led to underestimation of the proportions and NHS expenditure associated with discontinuations and residual need.

In addition, higher SES was associated with incomplete IOTN outcome fields among patients who completed treatment (but not among those who discontinued treatment), for reasons that are unclear. In contrast, lower SES was associated with missing outcome records. If missing outcome records were partly reflective of discontinuations (e.g., if dentists did not submit records because patients discontinued treatment and the dentists were initially unsure whether the patients would return), the association between lower SES and discontinuations would be attenuated.

Another limitation of the study relates to criticism regarding the ability of the IOTN to measure outcomes. The developers of the ICON argue against the use of the IOTN to investigate outcomes on the basis that it was ‘developed and validated to assess treatment entry and exits as separate phenomena, when they are clearly part of the same clinical process. This requires additional training and duplicates the effort of measuring what are often similar occlusal traits’ [35]. Nonetheless, the IOTN was used on the basis that it indicates the degree of residual normative need at the end of active treatment. Moreover, the IOTN remains the principle index used to assess individuals’ need for NHS orthodontic treatment need and the prevalence of malocclusion in the population [36, 37], and using the same index to establish both a baseline assessment of need and the treatment outcome is practical when assessing the effects of treatment. However, the IOTN outcome scores were measured at the end of active treatment, but there can be relapse after active treatment has finished [38], particularly if there is poor compliance with retention instructions. From this perspective, both the proportion of treatments that end with residual need and the expenditure on these treatments are underestimated. Lastly, the IOTN outcome scores were not independently validated, which may also have led to bias in the data, and thus an underestimation of residual need.

Regarding the implications of the findings, the quality of the NHS activity data could be increased by ensuring that outcomes are reported (and validated) for all treatments (except in cases where the patients fail to return, when only discontinuations, rather than IOTN scores, can be reported), and monitored by the service commissioners. This is in line with 2015 guidance on the delivery of NHS orthodontic care in Wales, which highlighted the importance of the development of local health board policies to ensure that treatment outcomes in terms of completions and discontinuations are reported for each patient [39]. Monitoring outcomes more closely could help service commissioners to determine which providers provide the best value for money.

In addition to recording information on discontinuations and IOTN outcome scores, there is a contractual requirement for dentists to monitor the outcomes of 20 patients plus 10% of the remainder of their patients using the PAR index [11], though these data are not collected using the NHS activity forms [12]. Implementing a contractual requirement to monitor the PAR scores of *consecutive* patients and to utilise independent third parties who are calibrated in the use of the index may help to reduce bias [15]. The British Orthodontic Society have noted that NHS commissioners may make participation in a peer review process a contractual requirement [15], and NHS guidance on commissioning has stated that ‘PAR scoring will in future be undertaken within a managed orthodontic clinical network…under a peer review mechanism’ [1].

Payment by Results (PbR) remuneration is used in many areas of the NHS [40], and could potentially help to improve orthodontic treatment outcomes [11]. However, a difficulty with implementing this policy is that there is a variation in the case mix (e.g., different percentages of patients from more deprived groups) between orthodontic clinicians, which would influence the percentages of patients who discontinue treatment and have residual need. Further, a PbR approach may generate perverse incentives for orthodontists to reject referrals of patients from disadvantaged backgrounds, widening inequalities further. In addition, although contract penalties for discontinuations may help to reduce the SES-related inequity in discontinuation, care would need to be taken to ensure that treatment was not continued in cases where there could be risk of harm to the patient (i.e., if compliance with oral hygiene advice was poor).

Key areas to be explored include how factors related to patient compliance underlie the associations between SES and orthodontic outcomes, and why higher SES was associated with incomplete IOTN outcome fields among patients who completed treatment.

## Conclusions

We found evidence of inefficiencies in the NHS orthodontic service, with 7.6% and 5.2% of NHS primary care orthodontic treatments in North West England ending in discontinuation and residual need, respectively. These outcomes were positively associated with lower SES, which raises concerns about inequity in the service and indicates that policies aimed at improving the levels of discontinuations and residual need may be more effective if they were targeted at patients from lower SES groups. In addition, 38.3% of treatments had missing outcome data, highlighting the need to improve the outcome monitoring systems.

## Additional file

Additional file 1: Percentage distribution/means of variables used in the adjusted treatment outcomes analyses, by socioeconomic status (SES). Additional file 1 contains information on the distribution of variables used to adjust for potential confounding: IOTN AC scores, gender, and age. This information is provided by IMD quintile. (DOCX 16 kb)

## Abbreviations

AC: Aesthetic Component
CI: Confidence interval
DHC: Dental Health Component
ICON: Index of Complexity, Outcome, and Need
IMD: Index of Multiple Deprivation
IOTN: Index of Orthodontic Treatment Need
LSOA: Lower-layer Super Output Area
NHS: National Health Service
NHSBSA: National Health Service Business Services Authority
OLS: Ordinary least squares
PAR: Peer Assessment Rating
PbR: Payment by Results
SES: Socioeconomic status
UK: United Kingdom
UOA: Unit of Orthodontic Activity
