# Supplementary material for: Socioeconomic disparities in orthodontic treatment outcomes and expenditure on orthodontics in England’s state-funded National Health Service: a retrospective observational study
Source: BMC Oral Health. 2017 Sep 19;17:123. doi: 10.1186/s12903-017-0414-1 (PMC5605975; doi:10.1186/s12903-017-0414-1)
Supplement: Additional file 1: — Percentage distribution/means of variables used in the adjusted treatment outcomes analyses, by socioeconomic status (SES). Additional file 1 contains information on the distribution of variables used to adjust for potential confounding: IOTN AC scores, gender, and age. This information is provided by IMD quintile. (DOCX 16 kb) [file 12903_2017_414_MOESM1_ESM.docx]

Percentage distribution/means of variables used in the adjusted treatment outcomes analyses, by socioeconomic status (SES)

| IMD quintile | Percentage distribution | | | | | | | | | |  |  | Mean |
| --- | --- | --- | --- | --- | --- | --- | --- | --- | --- | --- | --- | --- | --- |
|  | IOTN AC score | | | | | | | | | | Gender | | Age (years) |
|  | 1 | 2 | 3 | 4 | 5 | 6 | 7 | 8 | 9 | 10 | Male | Female |  |
| 1 | 0.2 | 0.7 | 3.3 | 7.1 | 6.8 | 25.1 | 23.7 | 24.0 | 6.7 | 2.2 | 41.4 | 58.6 | 13.3 |
| 2 | 0.0 | 0.7 | 4.2 | 8.7 | 7.0 | 27.1 | 21.0 | 22.4 | 7.3 | 1.8 | 43.5 | 56.5 | 13.2 |
| 3 | 0.2 | 1.1 | 4.3 | 8.4 | 8.0 | 29.8 | 21.3 | 20.3 | 5.7 | 1.1 | 42.6 | 57.4 | 13.1 |
| 4 | 0.1 | 0.4 | 2.9 | 9.8 | 7.2 | 28.6 | 22.1 | 20.1 | 7.9 | 0.9 | 43.9 | 56.1 | 13.1 |
| 5 | 0.2 | 0.6 | 4.8 | 8.9 | 6.6 | 26.3 | 24.2 | 19.6 | 7.0 | 1.8 | 44.9 | 55.1 | 12.9 |
| Missing | 0.0 | 0.4 | 3.6 | 5.4 | 4.8 | 34.6 | 20.8 | 23.2 | 5.4 | 1.8 | 43.3 | 56.7 | 13.1 |

AC, Aesthetic Component; IMD, Index of Multiple Deprivation; IOTN, Index of Orthodontic Treatment Need.
